# Permanent magnet array–driven navigation of wireless millirobots inside soft tissues

**DOI:** 10.1126/sciadv.abi8932

**Published:** 2021-10-20

**Authors:** Donghoon Son, Musab Cagri Ugurlu, Metin Sitti

**Affiliations:** 1 Physical Intelligence Department, Max Planck Institute for Intelligent Systems, 70569 Stuttgart, Germany.; 2 School of Mechanical Engineering, Pusan National University, 46241 Busan, South Korea.; 3 School of Medicine and College of Engineering, Koç University, 34450 Istanbul, Turkey.; 4 Institute for Biomedical Engineering, ETH Zürich, 8092 Zürich, Switzerland.

## Abstract

Creating wireless milliscale robots that navigate inside soft tissues of the human body for medical applications has been a challenge because of the limited onboard propulsion and powering capacity at small scale. Here, we propose around 100 permanent magnet array–based remotely propelled millirobot system that enables a cylindrical magnetic millirobot to navigate in soft tissues via continuous penetration. By creating a strong magnetic force trap with magnetic gradients on the order of 7 T/m inside a soft tissue, the robot is attracted to the center of the array even without active control. By combining the array with a motion stage and a fluoroscopic x-ray imaging system, the magnetic robot followed complex paths in an ex vivo porcine brain with extreme curvatures in sub-millimeter precision. This system enables future wireless medical millirobots that can deliver drugs; perform biopsy, hyperthermia, and cauterization; and stimulate neurons with small incisions in body tissues.

## Teaser

A wireless magnetic millirobot navigates inside an ex vivo porcine brain propelled by a permanent magnetic array–based force trap.

## INTRODUCTION

In the last decade, remarkable progress has been made to create small-scale medical robots that can operate inside the human body. The main benefit of such miniature robots is their small size, which does not require large openings to access many parts of our body invasively, minimizing surgical trauma and enabling new surgical procedures. However, many challenges also arise from their small size, due to the difficulty to place onboard robotic components, such as actuators, sensors, power source, computation, communication, and control electronics, into the tiny robot body (*1*). With these limitations, two main approaches have been used in the literature: tethered macroscale robots with small-scale end-effectors and untethered (wireless) small-scale robots. The former approach showed substantial improvement over the last decades. The laparoscopic robotic surgical systems (*2*, *3*), natural orifice transluminal endoscopic surgery (*4*–*8*), robotic capsule endoscopes (*9*–*12*), and steerable catheters and needles (*13*–*18*) have been studied rigorously, and some of them have become available already in hospitals. The latter approach has been progressing notably in the recent decade. Using remote actuation principles, such as magnetic fields, acoustic waves, and light, many wireless small-scale robots have been developed (*1*, *19*–*26*) for navigation and performing medical functions inside the body. Also, biohybrid microrobots have been proposed to enable cell-scale wireless robots by using the integrated live microorganisms as biological actuators, sensors, and taxis-based controllers (*27*–*29*). These small-scale robots have been demonstrated to navigate and operate inside body fluids and on body tissue surfaces; however, they have not shown the navigation inside body tissues due to the limited force and torque generated by the external actuation sources.

As the human body is composed of mostly soft tissues, enabling the small-scale mobile robots to navigate inside not only in fluids but also in soft tissues is important to access and treat diseases in some hard-to-reach parts of the human body. For example, a robot that can navigate inside brain could treat brain cancer without creating large surgical openings in skull and brain tissues. Recent attempts to enable remotely actuated miniature robots to navigate inside tissues have aimed for blood-clot removal (*30*–*33*), tissue drilling (*34*, *35*), and navigation in the brain (*36*, *37*). However, these previous studies are limited to a simple one-dimensional (1D) actuation and a low control and localization bandwidth with possible control instability issues caused by strong magnetic forces (*38*). Creating strong magnetic forces to penetrate tissues in a very controlled and stable manner has been the challenge in this field, especially due to the exponentially (on the order of 4) increasing magnetic forces on the robot toward the magnetic source, which is intrinsically unstable in the control aspect. This may result in the magnetic robot flying toward the magnetic source during a surgical operation when the localization and control systems are not extremely well designed with high bandwidths, which would be a high safety risk in medical magnetic robot systems.

**Fig. 1.**
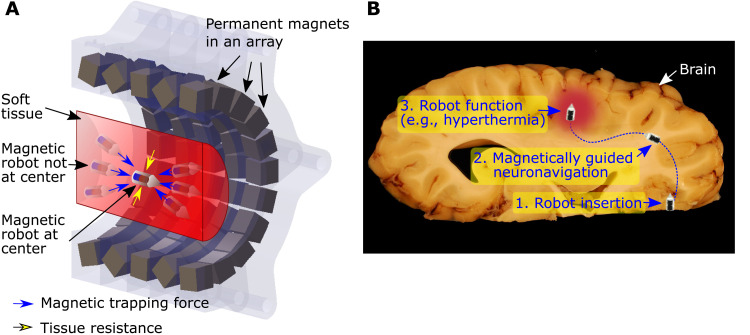
Permanent magnet array–based propelled and guided wireless magnetic millirobot for navigation inside body tissues via tissue penetration. (**A**) Schematic of the half-sectioned magnet array, where stable magnetic trapping forces always pull the robot in soft tissue to the center of the magnet array. (**B**) Application scenario of the proposed robotic tissue navigation system. The magnetic robot inserted to the edge of the brain or another soft tissue is guided by the magnet array–based trapping forces and torques, by following a planned 3D path to reach the final target destination. The robot could also perform various local and on-demand medical functions, such as drug delivery, hyperthermia, cauterization, biopsy, and neural stimulation, in the future. Figure from https://pixabay.com/photos/brain-anatomy-eyes-paerparat-horse-114071/, adapted by D.Son, Max Planck Institute for Intelligent Systems.

In this study, we propose to solve this challenge by creating a robot actuation system with a stable magnetic force trap to drive a wireless milliscale magnetic robot inside soft tissues. This system does not rely on a high-bandwidth localization and actuation feedback, which ensures safety in medical procedures. Moreover, this system can create very strong magnetic forces, which can enable locomotion inside soft tissues. It consists of an array of permanent magnets to generate a special magnetic field map that creates a magnetic force trap inspired by Halbach (*39*, *40*) and Aubert arrays (*41*–*43*), which have shown strong and uniform magnetic fields for nuclear magnetic resonance applications. Here, we propose a custom magnet array with 96 to 100 permanent magnets to create a stable magnetic force trap that can capture a magnetic robot at an area in 3D space leveraging mechanical interactions with a soft tissue at a point in 3D space with high magnetic gradients on the order of 7 T/m for driving it inside soft tissues. The magnet array generates a strong magnetic force toward the area of attraction in the array on the robot that deviates from the area of attraction (Fig. 1A). In addition, the induced magnetic torques align the robot’s orientation toward the center of the array, which facilitates the sliding motion of the robot toward the center. We created such a custom magnetic array by solving a nonlinear design optimization problem for creating the magnetic force trap based on a magnetic dipole model. Our experimental results show that this approach enables open-loop stability of magnetic millirobots in soft tissue phantoms and ex vivo porcine brain tissue. Furthermore, by combining the array with a robotic 3D positioning system, the path-following of complex trajectories in soft tissues are demonstrated in concert with visual monitoring using 2D video images or fluoroscopic x-ray medical images. In the future, the proposed design of the magnet array and robotic system under a fluoroscope could be used for creating a new generation of wireless medical millirobot systems for navigation and diverse medical functions, such as local and controlled drug delivery, cauterization, biopsy, and neural stimulation in brain and other soft tissues (Fig. 1B).

## RESULTS

**Fig. 2.**
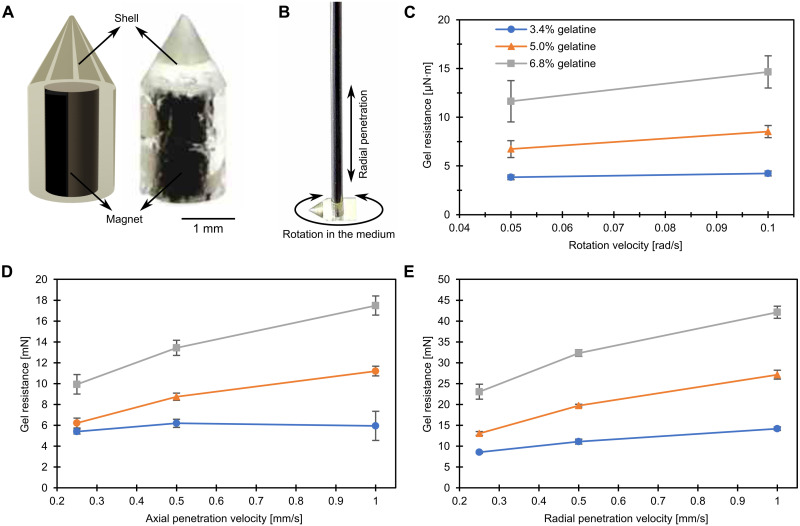
Design and experimental characterization of the magnetic millirobot inside a gelatin tissue phantom. (**A**) 3D computer-aided design (CAD) design and fabricated prototype of the robot. The robot is composed of a cylindrical NdFeB permanent magnet at the center and the conical tip cylindrical polymer housing. Photo credit: M. C. Ugurlu, Max Planck Institute for Intelligent Systems. (**B**) Three different direction schematics for characterizing the interaction between the robot and the gelatin soft tissue phantom. (**C**) Rotation resistance characterization results of the phantom with different gelatin densities. The resistance from the phantom shows a linear relationship with the angular velocity of the robot. (**D**) Axial penetration resistance results show a mostly linear relationship between the axial penetration speed and the resistance from the gelatin with different densities. (**E**) Radial penetration resistance results are similar to the axial penetration results with a linear relationship between the penetration speed and the resistance. Note that the radial penetration resistance is more than twice as high as the axial penetration one on average due to the large cross section from side than the top of the robot. Experimentally measured gel resistance values for robots with different cone angles and tip radii are given in fig. S1.

We designed and fabricated a cylindrical magnetic millirobot that can move inside soft tissues actuated by the external magnetic forces. The robot is composed of a cylindrical permanent magnet (1 mm in diameter and 2 mm in length) and a casing with a conical tip that covers the magnet (Fig. 2A). The radius of the robot’s conical tip is chosen as 50 μm to minimize the tissue damage during the penetration-based navigation (*44*). When the robot is placed inside the magnetic array (Fig. 1A), the magnetic forces generated by the array on the robot thrust the robot toward the magnetic center of the array. Undesired radial forces (compared to the axial forces) are canceled by the tissue resistance due to the high aspect ratio morphology of the robot where the large radial cross section creates the drag, while the pointy tip of the robot creates openings in the tissue with a smaller thrust force.

The robot-tissue interactions are characterized in terms of resistance (or drag) from the tissue expressed as force and torque when the robot moves at a specific velocity or rotational speed. The soft tissue phantom with multiple grades of gelatin (3.4, 5.1, and 6.8% by mass or weight) is used to mimic different soft tissues in the body. Here, 3.4 weight % (wt %) gelatin shows similar mechanical properties of a human brain in terms of penetration (*45*). The axial and radial resistance forces (with respect to the robot’s central axis) and the resistance torque (with respect to the axis perpendicular to the robot’s central axis; Fig. 2B) are measured in the gelatin matrices in the three grades with different penetration or rotational velocities (Fig. 2, C to E). For the axial penetration case (Fig. 2D), the resistance forces are measured between 5 and 6 mN when penetrating 3.4 wt % gelatin. At the slowest speed (0.25 mm/s) in 6.8 wt % gelatin, the gel resistance is measured as 9.9 mN. This shows that the array should create at least 10-mN axial pushing force for penetration. For the radial penetration case (Fig. 2E), the lowest value is measured as 8.5 mN. Therefore, to be able to restrain radial movements, the radial forces applied by the magnet array should be less than 8.5 mN. Comprehensive resistance measurement data with various robot geometries are shown in fig. S1.

**Fig. 3.**
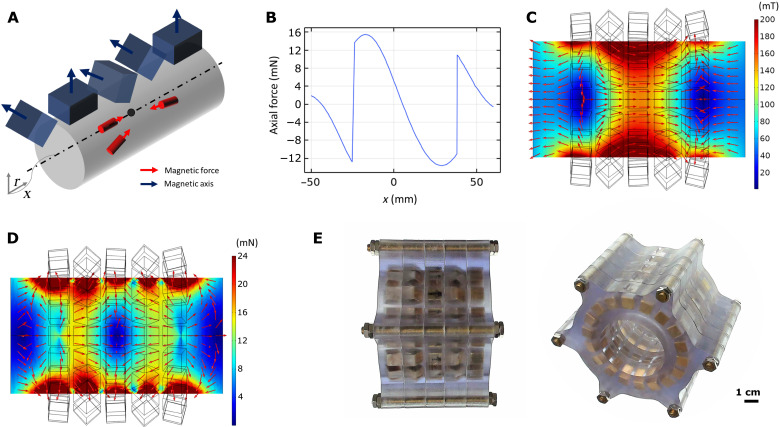
Design optimization of the magnetic array–based trapping system. (**A**) Schematic of the permanent magnet array design and desired forces when the robot is inside the workspace. The magnetic axes and the position of 100 permanent magnets are the parameters to optimize. (**B** to **D**) Simulation results of the final design using a magnetic dipole model. (B) The magnetic force profile on the center line shows that sufficient forces are achieved for tissue penetration exceeding 12 mN, and the force profile crosses the *x* axis showing that the force trap exists in the *x* axis. (C) Simulated 2D magnetic field profile shows that the magnetic field orients toward the center of the array with strong magnetic field flux densities in the workspace. (D) In simulated 2D magnetic force field (assumed that the robot orients to the direction of the magnetic field), a force trap in the *x* axis is seen around the center line, where forces lead to the center and at the center no force is applied to the robot. Note that the diverging radial forces are very small, which could be restrained by tissue resistance shown in Fig. 2E. (**E**) Side- and angled-view photos of the fabricated magnet array of the final optimal design. Geometric parameters of the final optimal design are given in fig. S2 and table S1. Photo credit: M. C. Ugurlu, Max Planck Institute for Intelligent Systems.

We designed and fabricated the robot actuation system, which can drive the magnetic robot in soft tissues with a continuous penetration by creating a strong magnetic force and torque trap. Inspired by Halbach (*39*, *40*) and Aubert (*41*–*43*) arrays, a permanent magnet array gives much higher design space compared to previous magnetic robot actuation systems based on 8 to 15 magnetic sources (*12*, *46*–*53*). Creating the permanent magnet array was formulated in a design optimization problem. Here, our design goal is to induce a magnetic force larger than 12 mN (Fig. 2D) on the robot, which has a footprint smaller than 3 mm in length and 1.5 mm in diameter in the workspace. Another critical goal is to create a stable force trap in the workspace so that the robot automatically centers in an open-loop manner (Fig. 3A). This is crucial because inducing a very strong magnetic force often creates a magnetic runaway problem, where the robot shoots to one of the magnetic sources, which can damage the tissue in an uncontrolled manner. This design goal was achieved by optimizing the configuration of the permanent magnet array (the magnetic axes and positions of magnets of Fig. 3A) to create a force profile that crosses the *x* axis (Fig. 3B) while minimizing the radial magnetic force escaping from the central axis of the array (Fig. 3D). In addition, it is beneficial to create the magnetic field, which orients toward the center of the array to make the robot head toward the center of the array (Fig. 3C). We formulated the optimization goal and the design constraints in a nonlinear optimization routine. The optimization problem was solved by a numerical nonlinear optimization solver using the magnetic dipole model (see Materials and Methods for the details of the formulation). Because the workspace is far enough from the permanent magnets (outside of 1.5 radius sphere of each permanent magnet), the magnetic field estimated by the magnetic dipole model has more than 99% accuracy (*54*).

The final design from the optimization results is shown in Fig. 3E. The array was fabricated using 3D printing of the array housing from the final design and embedding permanent magnets manually. The magnetic simulations confirm the force trap, which crosses the *x* axis and the strongest magnetic force exceeds 12 mN in the axial direction (Fig. 3B), magnetic field toward the area of attraction (Fig. 3C), and the weak radial magnetic force (Fig. 3D), which does not exceed the tissue resistance (Fig. 2E). The experimental measurement of the magnetic fields of the fabricated custom magnet array is shown in fig. S3.

**Fig. 4.**
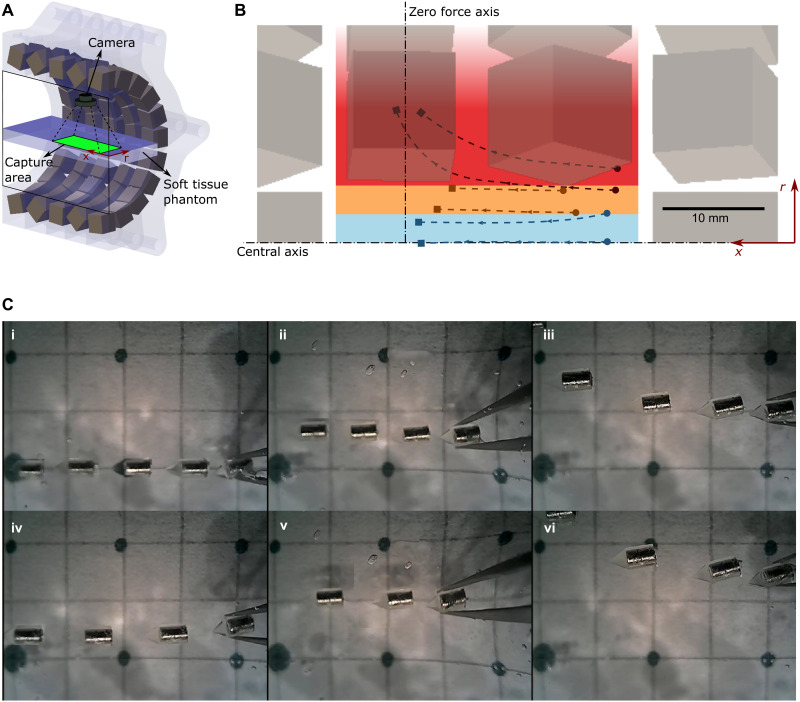
Open-loop stability demonstration of the magnetic millirobot trapping. (**A**) Half-sectioned schematic of the trajectory measurement experiments. The robot is inserted in the soft tissue phantom, while its trajectory in the image area is recorded by a top-view camera. (**B** and **C**) Paths followed by the robot from different insertion points (●) to final positions (■). Colored regions indicate stable [blue in (B), (i) and (iv) in (C), 0 < *r* < 3 mm], marginally stable [orange in (B), (ii) and (v) in (C), 3 mm < *r* < 6 mm], and diverging [red in (B), (iii) and (vi) in (C), *r* > 6 mm] regions (see movie S1). Considering the workspace as a bore radius of 30 mm, the stable and marginally stable regions cover 20% of the workspace. Photo credit: M. C. Ugurlu, Max Planck Institute for Intelligent Systems.

We first tested the performance of the magnetic force trap created by the array on the magnetic robot in soft tissue phantoms. Soft tissue phantoms with multiple grades (3.4, 5.1, and 6.8 wt %) were fabricated to mimic different soft tissues in the human body. The robot and the soft tissue phantom were placed inside the array, and the trajectory of the robot was recorded by the camera installed in the array (Fig. 4 and movie S1). The trajectory measurement experiment revealed the open-loop stability of the magnetic array and the robot. When the robot was in the stable zone (blue color in Fig. 4B), the robot moved toward the magnetic center of the array. When the robot was placed in the marginally stable zone (orange color in Fig. 4B), the robot did not center or diverge. When the robot was placed in the diverging zone (red color in Fig. 4B), the robot diverged and run away toward one of the permanent magnets in the array. Considering the workspace as a bore radius of 30 mm, the stable and marginally stable regions cover 20% of the workspace. This result shows the open-loop stability and suggests a control strategy, where the robot is placed inside the stable zone and can navigate in a stable manner in the soft tissue by relatively changing its 3D position with respect to the array. Further, we simulate the trajectory of the robot in terms of the tissue resistance to check how the regions of stability changes (fig. S4, simulation details in Supplementary Text). Our simulation results suggest that the stable region can be expanded by increasing the radial drag coefficient, *c*_r_, and decreasing the axial drag coefficient, *c*_a_, of the robot in the tissue. In addition, small radial drag coefficient and large axial drag coefficient can make the stable region very small so that the robot can become easily unstable. Here, the drag coefficients are the functions of the robot shape and the surrounding tissue, which should be carefully chosen to maximize the stable region by increasing the radial drag and by minimizing the axial drag. Note that these drag coefficients could be experimentally measured by calculating the slope of resistance-penetration speed graphs (drag coefficients of the current robot shape with 3.4 wt % gelatin matrix: *c*_a_ = 3.4 mN·s/mm, *c*_r_ = 10.9 mN·s/mm; Fig. 2).

**Fig. 5.**
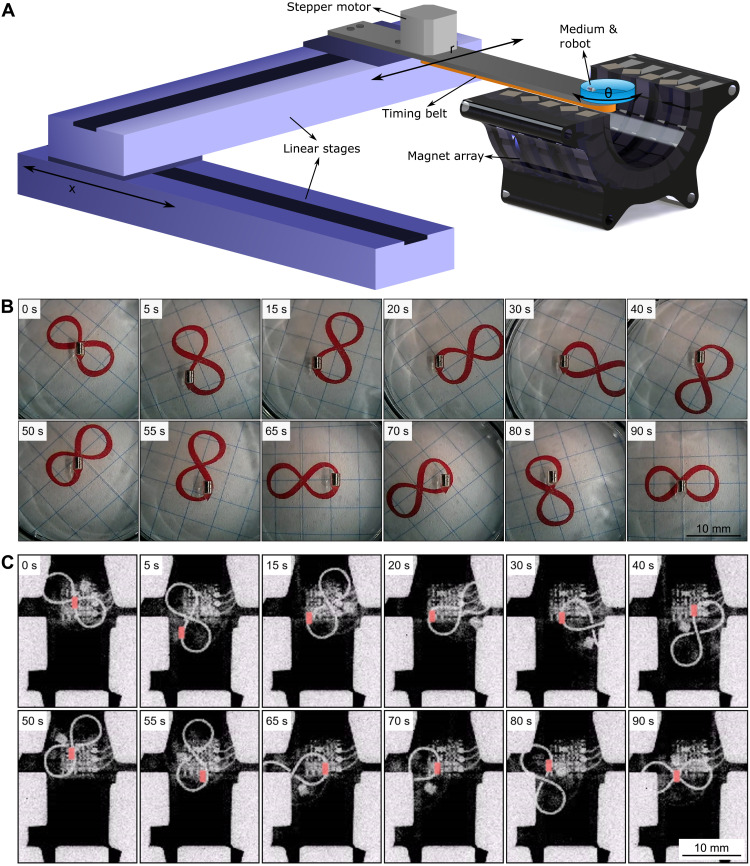
Experimental 2D path-following demonstration of the robot navigating inside a gelatin soft tissue phantom. (**A**) Schematic of the path-following experimental setup with the linear and rotational motion stage, half-section of the magnet array, robot, and tissue phantom (medium). While the linear stages control the axial (*x*) and radial (*r*) positions, the stepper motor rotates the phantom via a timing belt (θ). (**B**) Video snapshots from the top-view camera from a complete path-following experiment on an ∞-shaped path in the soft tissue phantom. (**C**) X-ray fluoroscopy video snapshots of the same path-following experiment. Robot is colored in red to make it distinguishable (see movie S2). The ∞-shaped path made out of lead is placed under the petri dish for easy fluoroscope path imaging. Photo credit: M. C. Ugurlu, Max Planck Institute for Intelligent Systems.

For the active navigation of the robot in soft tissues, the array was combined with a 3D robotic motion system, which translated and rotated the soft tissue relative to the array (Fig. 5A). Here, the array stayed still, and the magnetic robot embedded gelatin medium changed its position and orientation relative to the array. This enabled the robot’s navigation in gelatin relative to the coordinate frame of the gelatin. Furthermore, the system was combined with a fluoroscopic x-ray medical imaging system to demonstrate the compatibility with clinical imaging. Here, we removed a couple of permanent magnets in the array to secure the x-ray imaging pathway (fig. S5). Therefore, 4 magnets were taken out from the 100 magnet array to enable imaging access at the center with minimal change in magnetic trapping and guiding. To show the paths in x-ray images clearly, π-shaped and ∞-shaped lead wires were placed under the soft tissue phantom (gelatin 3.4 wt %).

By observing the robot and the medium from the top-view camera and fluoroscope, the relative position changes induced the motion of the robot in the gelatin medium. The robot followed the π-shaped and ∞-shaped paths (Fig. 5, B and C, and movie S2). Although these paths were challenging due to their extreme curvatures and complex shapes, the robot could still follow them in a stable manner. Here, the worst-case path deviation error was 0.47 ± 0.30 mm in the second trial of the ∞-shaped path (table S2). Note that these complex paths are not possible in steerable needle approaches due to the extreme curvatures. Here, the robot was controlled by placing it in the stable zone (Fig. 4C) while changing its relative axial position and orientation, which created the robot’s motion toward the center of the array by solving inverse kinematics of the robot arm.

**Fig. 6.**
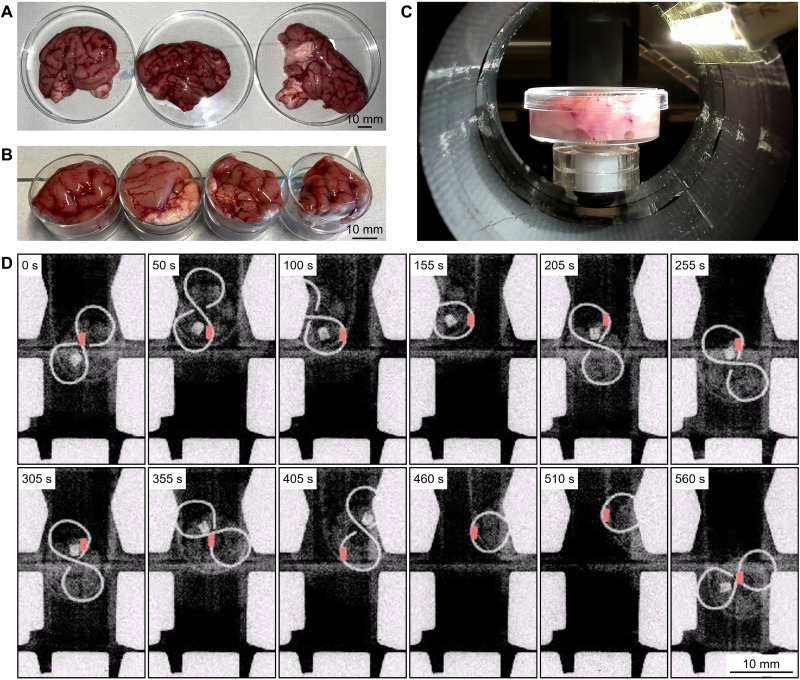
Ex vivo 2D path-following experiments in a fresh porcine brain. (**A** and **B**) Prepared fresh porcine quarter-cut (B) and whole (A) brain tissues. (**C**) Cut piece of a porcine brain attached to the robotic motion stage during the ex vivo path-following experiments. (**D**) X-ray fluoroscopy image snapshots of a complete path-following experiment on an ∞-shaped path in the porcine brain (see movie S3). Robot is colored in red to make it distinguishable, where the desired path is visible in the x-ray images using a copper wire bent in ∞-shape and located under the brain tissue. π-shaped path-following experimental results are available in fig. S6. Photo credit: Musab Cagri Ugurlu, Physical Intelligence Department, Max Planck Institute for Intelligent Systems.

Last, the robot was tested in a porcine brain ex vivo. A quarter-cut and whole fresh porcine brains (Fig. 6, A and B) were placed in a 33-mm-diameter petri dish connected to the robotic motion system. Unlike the previous experiments in the transparent soft tissue phantoms, the brain tissue is nontransparent and its stiffness is nonhomogeneous, which made it more challenging. Here, the localization of the robot was solely conducted by the x-ray fluoroscopy. The path made of the lead wire was placed under the petri dish to make the path to be visualized easily under the fluoroscope. The robot navigated inside the brain guided by fluoroscopy with stable motions (Fig. 6D and movie S3). Because of the nonhomogeneity of the brain, the robot had different traveling speeds in different regions of the brain. Although the various stiffness of the brain is not measured and registered before the experiment, the stable navigation was still achieved because of the open-loop stability of the magnetic array and the robot. We conducted a total of four experiments to follow two different types of paths (∞-shaped path in Fig. 6D and π-shaped path in fig. S6). The experiments showed a path error of 0.30 ± 0.27 mm in overall experiments (table S3, path deviation error). Each path-following experiment took approximately 4 to 8 min (see movie S3). Some parts of the brain were much stiffer, where the use of the maximum magnetic field gradient was used to penetrate the stiffer tissue (placing the robot far from the magnetic center in the stable region in Fig. 4B). In all ex vivo path-following experiments, the robot showed a stable navigation.

## DISCUSSION

While the proposed new magnetic actuation system can stabilize and navigate a magnetic millirobot inside soft tissues under fluoroscopy imaging, the current system prototype has some limitations. First, the current workspace of the array is relatively small for operating inside large animals and humans. The array has a 6 cm diameter, which is suitable for small animals, such as mice, rats, and rabbits. An adult human head is around 20 cm diameter, which requires at least five times upscaling of the array with the marginal spaces to move and rotate the array around the head. Because of the relationship between the magnetic force and the distance, the force applied to the robot will drop to one-fifth of the current design (fig. S7). This can be problematic to penetrate relatively stiff regions of the brain tissue, and in the worst case, the robot can be stuck in such tissues. However, this issue could be resolved by adding extra layers of permanent magnets to the current array system to augment the magnetic forces further. As an alternative, the permanent magnets could also be replaced by electromagnets composed of superconducting materials to boost the magnetic strength, as commonly implemented in magnetic resonance imaging systems.

Further miniaturization of the robot would reduce the tissue damage caused by the robot during penetration. As the magnetic force scales by the volume of the robot (proportional to the magnetic material volume of the robot) while the tissue resistance scales by the area of the robot, the volume reduction of the robot reduces the effective force to penetrate the tissues compared to the tissue resistance. Thus, there is a miniaturization limit of the robot to access tissues with strong resistance for the given magnetic actuation system. This limit could be reduced notably by enhancing the actuation system as mentioned above, by means of multiple layers of the magnetic array or electromagnets with superconducting materials.

The current robotic system is not patient oriented yet. Here, the system assumes that the magnetic array stays static, while the brain rotates around for the navigation of the magnetic robot in the tissue. This can cause discomfort to patients due to the motion created by the moving stage where the patient would lie. Ideally, the magnetic array should move around while the patient stays static to secure the safety and comfort of the patient. This can be achieved by creating a robotic system that can rotate and translate the magnetic array in five degrees of freedom (DOFs) around the patient, as a future work.

As other future works, first, the autonomous control and automatic path-following need to be implemented under 2D fluoroscopy imaging (with a prior 3D high-resolution map of the workspace by magnetic resonance imaging or computer tomography) for semi-automatic guidance. In the current study, a trained user controls the moving stage with visual feedback from the images from the camera or the fluoroscope. This process could be automated using computer vision and control techniques to localize the robot and guide the robot from an initial point to an endpoint following a planned path. This can help medical doctors plan and perform the surgery seamlessly with the help of easy-to-use robotic planning and guiding system. Second, diverse medical functions could be added on the robot for practical use. Although the current version of the magnetic robot system only shows the soft tissue navigation, the same system could also have various medical functions, such as local on-demand drug release, hyperthermia, biopsy, and neural stimulation by integrating drugs to the robot body with a triggered release mechanism (*55*), a metallic layer on the surface with a radio frequency or eddy current-based controlled remote heating (*56*), a remotely triggered biopsy microgripper (*57*), and a magnetopiezoelectric nanoparticle or nanofilm coating on the surface (*58*), respectively. We envision that such multifunctional wireless millirobots could enable access to complex tight regions inside tissues and reduce the risk of surgical complications and traumas. They can even stay inside the tissues for a desired duration and repeat the same medical procedures if the disease repeats, as an implantable medical device.

## MATERIALS AND METHODS

### Fabrication of the magnetic millirobot

The robot consists of a shell and a cylindrical NdFeB N52 grade magnet (Product number: 3424, EarthMag GmbH, Dortmund, Germany) (Fig. 2A). The polymeric robot shell was directly printed using a 3D microprinter based on two-photon polymerization (Photonic Professional GT, Nanoscribe GmbH, Karlsruhe, Germany) using IP-Q photoresist (Nanoscribe GmbH, Karlsruhe, Germany). The magnet with 1 mm diameter and 2 mm height (Webcraft GmbH, Gottmadingen, Germany) was manually inserted in this shell.

### Design optimization of the magnetic array

The permanent magnet array consisting of NdFeB N52 grade magnet cubes magnet (Product number: 3339, EarthMag GmbH, Dortmund, Germany) is designed to create a strong magnetic force trap at an area in the space. This design optimization problem is formulated on the basis of the magnetic dipole model (*46*). The magnetic field created by a magnetic dipole is

$$b=\frac{\mu_{0}}{4\pi {\left\|r\right\|}^{3}}\;(3\hat{r}{\hat{r}}^{T}-I)m$$

where **b ∈** ℝ^3^ is the magnetic field, μ_0_ is the permeability of free space, **r** ∈ ℝ^3^ is the displacement vector of the point of interest from the center of the magnetic source, $\hat{\cdot }$ is the operator of vector normalization, **I** is the three-by-three identity matrix, and **m** ∈ ℝ^3^ is the magnetic moment of the magnetic source. The magnetic force created on another magnetic source is

$$f=\frac{3\mu_{0}}{4\pi {\left\|r\right\|}^{4}}(({\hat{\;r}}^{T}m_{r})m+({\hat{\;r}}^{T}m)m_{r}+(m_{r}^{T}m-5(r^{T}m)(r^{T}m_{r}))\hat{r})$$

when the magnetic robot with magnetic moment **m**_r_ ∈ ℝ^3^ is located at **r**.

To create a magnetic force trap at the central axis of the array, we define a force profile as expressed in Fig. 3B. This force profile shows at least 12 mN of the magnetic force for the tissue penetration and crossing on the *x* axis for the force trap where the robot is automatically stabilized. This force profile becomes one of the constraints of the design optimization problem. In addition, we define other constraints to restrict the radial force so that the robot does not drift away from the central axis with the help of tissue resistance. The optimization goal here is to maximize the axial magnetic force. The configuration of the permanent magnets (e.g., the positions and orientations of the magnets) becomes the parameters for the optimization. All of this could be formulated in a nonlinear optimization routine as

$$x^{*}=\text{arg}\;\underset{x}{\text{min}}-{|f_{a}(p_{1})|}^{2}$$

subject to

$$f_{a}(p_{1})>12\;\text{mN}$$

$$f_{a}(p_{2})<0\;\text{mN}$$

$$\text{max}(|f_{r}(p_{3})|)<5\;\text{mN}$$

$$\frac{df_{a}(p_{4})}{\mathrm{dx}}<0$$

where *f_a_*(**p**) is the axial force evaluated at a point **p**, *f_r_*(**p**) is the radial force evaluated at a point **p**, **p**_1_ is the point on the axis in the workspace where the maximum force is intended (e.g., 20 mm away from the center of the array), **p**_2_ is the point on the other side of the axis restricting the force profile to cross the *x* axis, **p**_3_ is the concatenated position vectors to represent distributed points (every point in 2-mm grid) in the workspace to evaluate the multiple radial forces, **x** is the configuration of the permanent magnets including their positions and orientations assuming axisymmetric, and **p**_4_ is the concatenated points on the central axis of the array. Here, differentiation is forced to be negative to force the monotonically decreasing force profile as shown in Fig. 3B. As a nonlinear solver, the interior point algorithm programmed in a commercial computing language (fmincon.m in MATLAB, MathWorks Inc., MA, USA) is used to solve the formulated design optimization problem. The problem was solved within 150 iterations, resulting in the configuration shown in Fig. 3E. Although the optimization is performed locally, the optimization converged to the final solution even with multiple random initial parameters, implying that the optimization is close to the global optimum.

The final design of the array is verified by a commercial finite element analysis magnetic simulation tool (COMSOL Multiphysics 5.4, COMSOL Inc., Stockholm, Sweden) by placing the permanent magnets in the configuration achieved from the design optimization.

### Robotic motion stage

The robotic stage changes the position and orientation of the soft tissue phantom (or the porcine ex vivo brain) inside the magnet array. The stage consists of two linear axes and a rotational axis. The linear axes move perpendicular to each other on the horizontal plane and define the axial and radial position (*x* axis and *r* axis) inside the array. The rotational axis is attached on the second linear axis. It rotates the soft tissue phantom (or the porcine brain) about the vertical *z* axis and provides change of direction of the robot (θ) inside the medium. In our setup, we used two linear translation stages (model: LTS300/M, Thorlabs Inc., Newton, NJ, USA). The soft tissue phantom or the porcine brain samples were placed on a carriage, which is connected to a stepper motor (model: NEMA17-01, Neukirchen-Vluyn, Germany) via a timing belt to actuate the rotational axis. The overall system was driven by Robot Operating System (ROS Melodic), which runs in Linux operating system (Ubuntu 18.04). The operator modulated the control inputs through a wireless gamepad (model: F710, Logitech, Newark CA, USA) while the operator was getting the visual feedback either from images from a mini camera (model: YC225-P/FBA, CORPRIT, China) or fluoroscopic x-ray images. A cabinet x-ray system (model: XPERT 80, KUBTEC, Stratford CT, USA) is used for imaging of the ex vivo path-following experiments. The device captures images inside the cabinet on a 20 × 20 cm area. During the experiments, the x-ray accelerating voltage is set to 90 kV and the images are recorded at 25 frames per second.

### Motion control

In this study, the navigation system is demonstrated on a 2D workspace where three independent motions—axial translation (*x* axis), radial translation (*r* axis), and rotation (θ)—are defined. These motions are driven by the 3-DOF stage (Fig. 5A). The user adjusts *r*-position to keep the robot close to the central axis. Therefore, it remains to be in the stable region (Fig. 4B). While the robot is in the stable region, adjusting the *x*-position results in a change of the thrust force, which is characterized by the simulations and measurements (Fig. 3B). If the robot is moved further away from the center, the array applies a higher thrust force toward the magnetic center of the array. Therefore, the user can use *x*-position to adjust the magnitude of the thrust force. Last, to follow curvatures and make turns, applying rotation results in a respective change of the orientation of the robot due to the alignment by the magnetic field.

### Fabrication of the soft tissue phantom

Gelatin matrices are used to build the phantoms in robot-gelatin interaction experiments, open-loop stability tests, and path-following tests. The robot-gelatin interaction experiments and open-loop stability tests used three different densities (3.4, 5.1, and 6.8 wt %) using bovine gelatin powder (CAS Number: G9382, Merck KGaA, Darmstadt, Germany) for mimicking different strengths of soft tissues. Gelatin matrices are prepared in the procedure of weighing the powder and the water, heating up the water to 90°C, mixing the powder and the water mixture, and cooling to room temperature so that the gelatin solidifies as a matrix.

### Preparation of ex vivo porcine brains

For the ex vivo experiments, fresh porcine brains from a local butcher are used. The size of a single brain was around 80 mm in diameter, which are cut into four pieces to place in a petri dish with a 33 mm diameter (Fig. 6, A and B). To see the path inside the x-ray machine, a path made out of a lead wire is attached underneath the petri dishes to be seen in fluoroscopic images. Last, the robot is pushed inside the brain sample at the edge of the petri dish with a tweezer before the path-following test starts.

## Notes

View/request a protocol for this paper from *Bio-protocol*.
