# Supplementary material for: Permanent magnet array–driven navigation of wireless millirobots inside soft tissues
Source: Sci Adv. 2021 Oct 20;7(43):eabi8932. doi: 10.1126/sciadv.abi8932 (PMC8528412; doi:10.1126/sciadv.abi8932)
Supplement: Supplementary file 1 — Supplementary Text Figs. S1 to S7 Tables S1 to S3 Legends for movies S1 to S3 [file sciadv.abi8932_sm.pdf]

Science Advances
AAAS

Supplementary Materials for

Permanent magnet array–driven navigation of wireless millirobots inside soft tissues

Donghoon Son, Musab Cagri Ugurlu, Metin Sitti*

*Corresponding author. Email: sitti@is.mpg.de

Published 20 October 2021, *Sci. Adv.* **7**, eabi8932 (2021)
DOI: 10.1126/sciadv.abi8932

The PDF file includes:

Supplementary Text
Figs. S1 to S7
Tables S1 to S3
Legends for movies S1 to S3

Other Supplementary Material for this manuscript includes the following:

Movies S1 to S3

Supplementary Text. Robot Trajectory Simulation Method

For simulating the trajectory of the robot, it is assumed that the inertial effect is negligible due to the small mass of the robot and the large drag from the surrounding tissue. The Newton's law during a quasistatic motion becomes

$$\mathbf{f}_{\mathrm{m}} - \mathbf{c} \circ \mathbf{v} = m\mathbf{a} \approx 0$$

where $\mathbf{f}_{\mathrm{m}}$ is the magnetic force on the robot, **c** is the drag coefficients vector, **v** is the speed of the robot, m is the mass of the robot, $\circ$ is the elementwise multiplication, and **a** is the acceleration of the robot. By assuming that the robot instantaneously aligns to the external magnetic field because the magnetic torque is stronger by an order of magnitude than the rotational tissue resistance, the magnetic force becomes a function of the position of the robot as

$$\mathbf{f}_{\mathrm{m}} = \mathbf{f}_{\mathrm{m}}(\mathbf{x})$$

where **x** is the position of the robot. This mapping is achieved by interpolating the magnetic force map (**Fig. S3**B). For simplicity, the robot's motion can be decomposed into axial and radial directions of the robot. The axial directional unit vector is achieved by normalizing the external magnetic field as

$$\hat{\mathbf{n}} = \frac{\mathbf{b}(\mathbf{x})}{|\mathbf{b}(\mathbf{x})|}\,.$$

The magnetic force in the axial direction is

$$\mathbf{f}_{\mathrm{m}\parallel} = (\mathbf{f}_{\mathrm{m}} \cdot \hat{\mathbf{n}})\hat{\mathbf{n}}$$

and the radial direction is

$$\mathbf{f}_{\mathrm{m}\perp} = \mathbf{f}_{\mathrm{m}} - \mathbf{f}_{\mathrm{m}\parallel}\,.$$

The velocity in each axis can be represented as

$$\frac{\mathrm{d}\mathbf{x}_{\parallel}}{\mathrm{d}t} = \frac{1}{c_{\mathrm{a}}}\mathbf{f}_{\mathrm{m}\parallel} \quad \text{and} \quad \frac{\mathrm{d}\mathbf{x}_{\perp}}{\mathrm{d}t} = \frac{1}{c_{\mathrm{r}}}\mathbf{f}_{\mathrm{m}\perp}$$

where $\mathbf{x}_{\parallel}$ and $\mathbf{x}_{\perp}$ are used to represent position changes of the robot in axial and radial direction for the differentiation, and c_{a} and c_{r} are drag coefficients of the robot in the tissue, respectively. Thus, the trajectory can be calculated by integrating the derivatives of the robot positions as

$$\mathbf{x}(t) = \mathbf{x}(0) + \int_{\tau=t_0}^{\tau=t} \frac{1}{c_{\mathrm{a}}}\mathbf{f}_{\mathrm{m}\parallel}\big(\mathbf{x}(t)\big) + \frac{1}{c_{\mathrm{r}}}\mathbf{f}_{\mathrm{m}\perp}\big(\mathbf{x}(t)\big)\,\mathrm{d}\tau$$

where $\mathbf{f}_{\mathrm{m}\parallel}(\cdot)$ and $\mathbf{f}_{\mathrm{m}\perp}(\cdot)$ indicate that they are function of the trajectory, $\mathbf{x}(t)$. This is solved numerically by a custom-made simple integration function in a commercial programming language (MatLab, Mathworks, Inc., Massachusetts, United States). The integration process could be early terminated when the speed of the robot is smaller than a preset value (0.2 mm/s) assuming that the robot is stuck in the tissue.

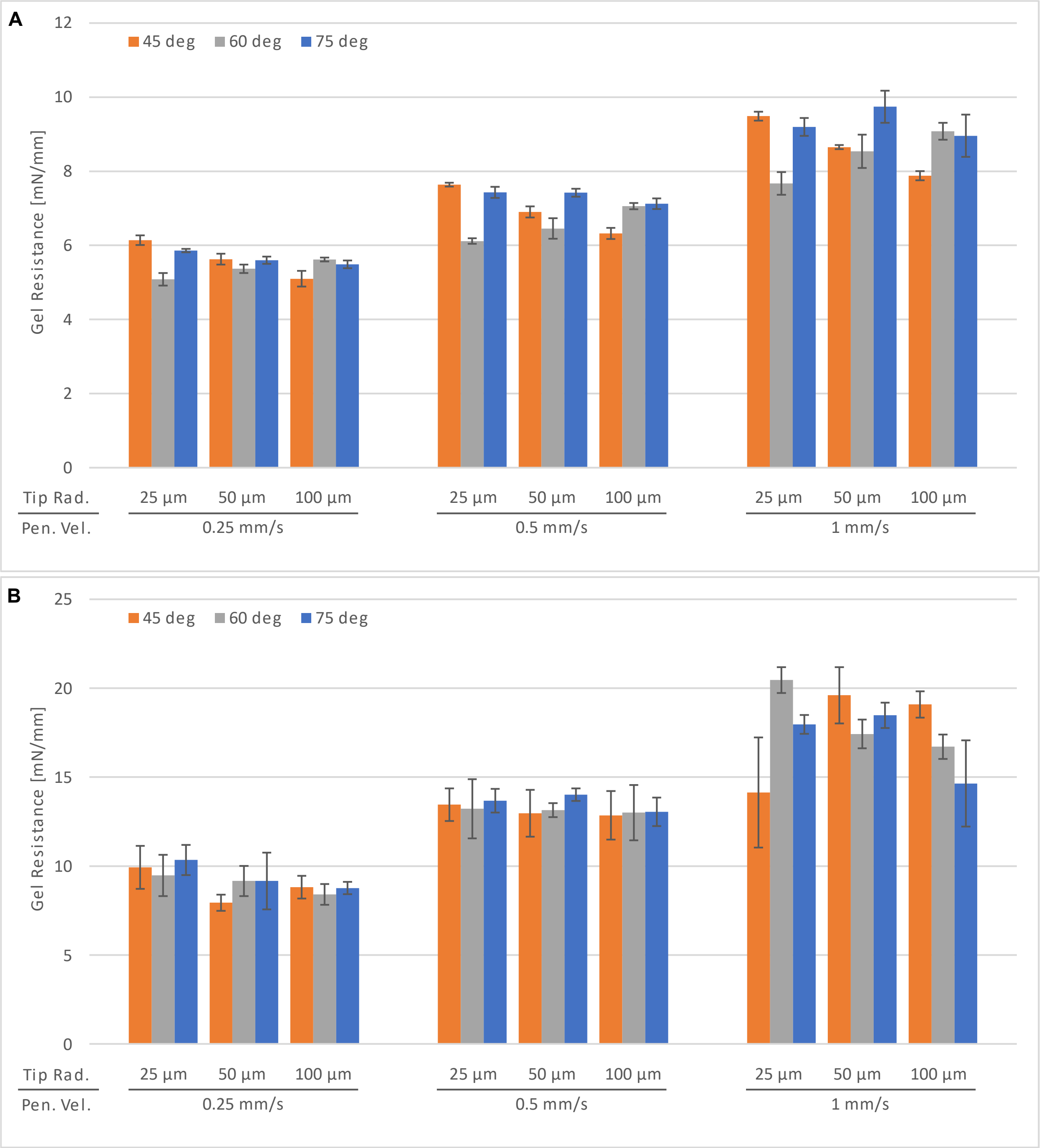

Fig. S1. Experimental characterization of the magnetic millirobot inside a gelatin tissue phantom. (**A**) Axial penetration resistance results for different tip radius, cone angle and penetration velocity values. (**B**) Radial penetration resistance for different tip radii, cone angles and penetration speeds. In both graphs, nine adjacent bars share the same penetration velocity while three adjoint bars has the same tip radius but different cone angles according to the colour legend. The error bar shows the standard deviation of 5 measurements for each shape, speed, and direction.

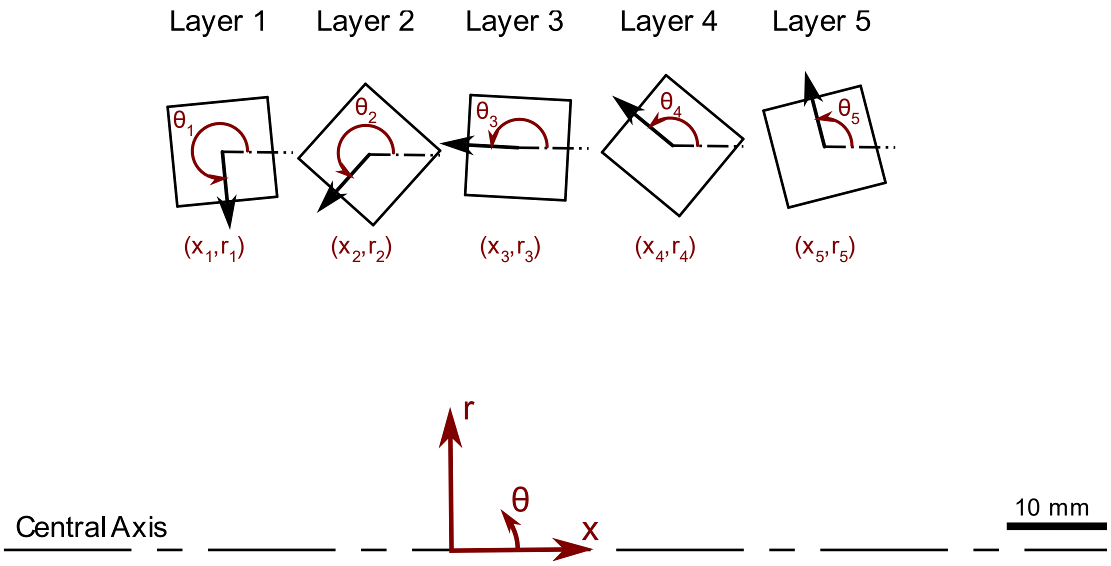

Fig. S2. Sketch of the individual magnet positions and orientations in the optimized magnet array design. (x_i, r_i) indicates the *x*- and *r*-coordinates of the geometric center of the cubic magnet and θ_i is the angle between the magnetic moment of the magnet and *x*-axis. The values of the corresponding parameters are given in table S1.

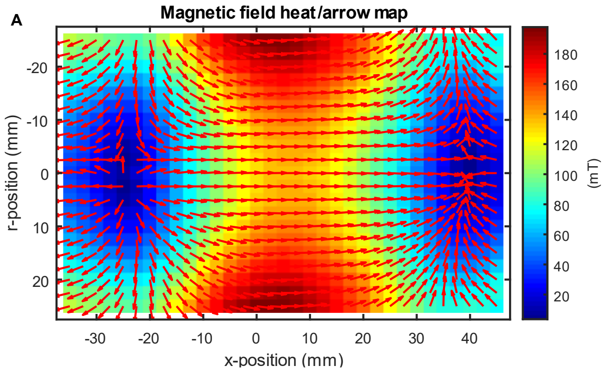

Fig. S3. Experimental magnetic field and calculated magnetic force maps of the magnet array in the *xr*-plane. The experimentally measured magnetic field (**A**) and the calculated magnetic force field (**B**) from the measured magnetic field data assuming that the magnetic robot orients in the same direction of the magnetic field at a point (compare with Fig. 3C,D).

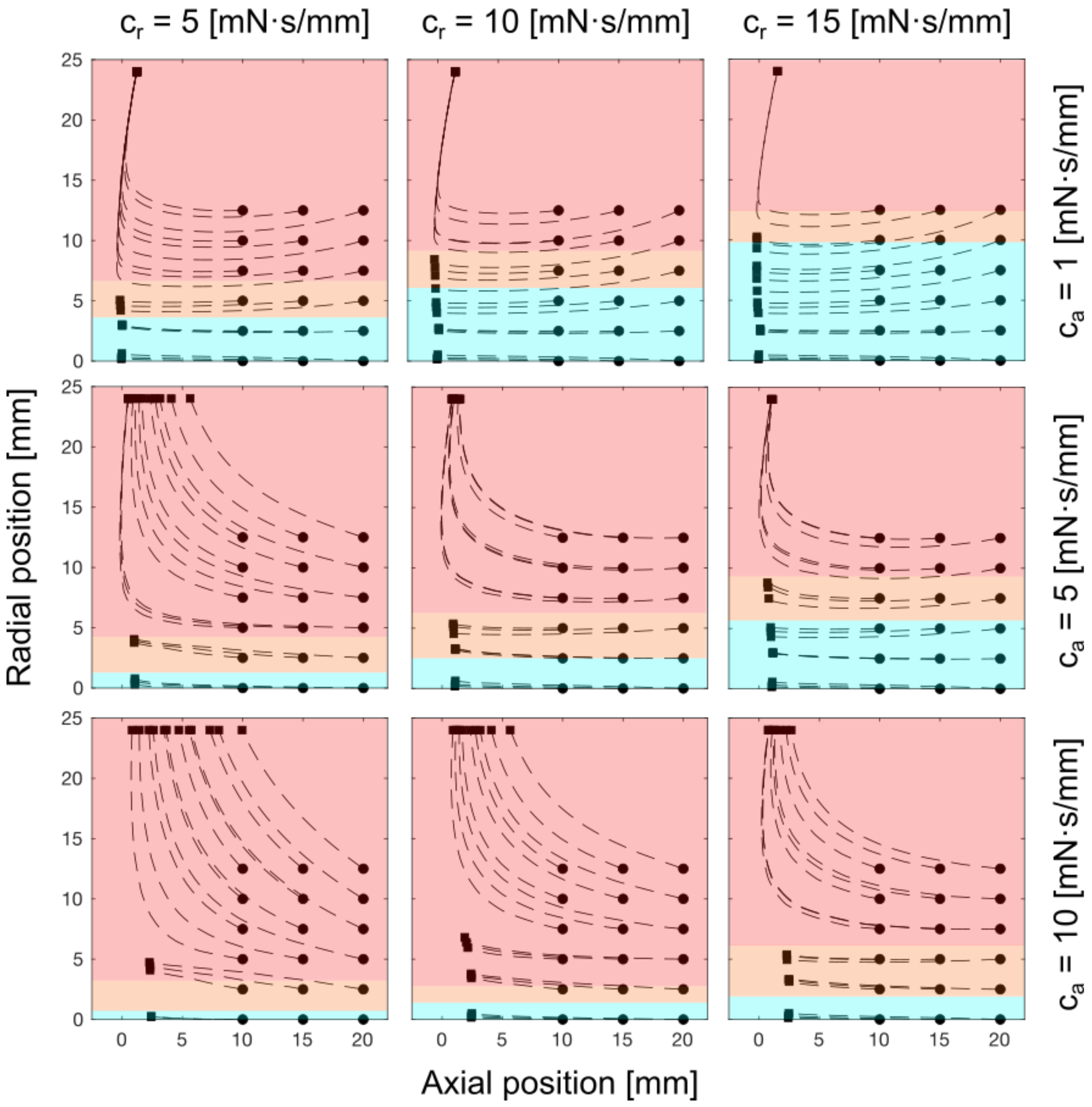

Fig. S4. Simulated trajectories in terms of the axial drag coefficient, c_a, and the radial drag coefficient, c_r, with respect to the robot's central axis. The stable zone increases by decreasing c_a and increasing c_r. The central figure represents the trajectories with drag coefficients of the current experimental setup (experimental drag coefficients: c_a = 3.4 mN·s/mm, c_r = 10.9 mN·s/mm). Path followed by the robot from different starting points (●) to final positions (■). Colored regions indicate stable (blue), marginally stable (orange), and diverging regions (red).

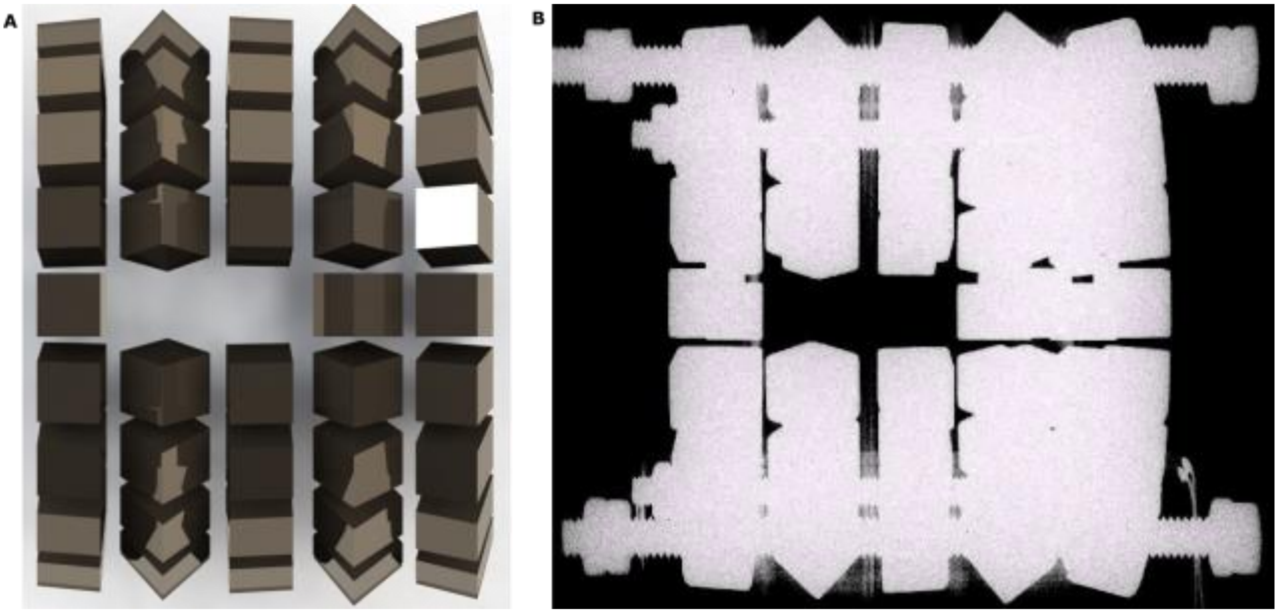

Fig. S5. Modified version of the magnet array that has the X-ray imaging pathway. (**A**) 3D CAD drawing of the 100 magnet array after removing 4 permanent magnets to secure the X-ray imaging pathway at the center. (**B**) X-ray image of the magnet array inside the X-ray fluoroscopy system.

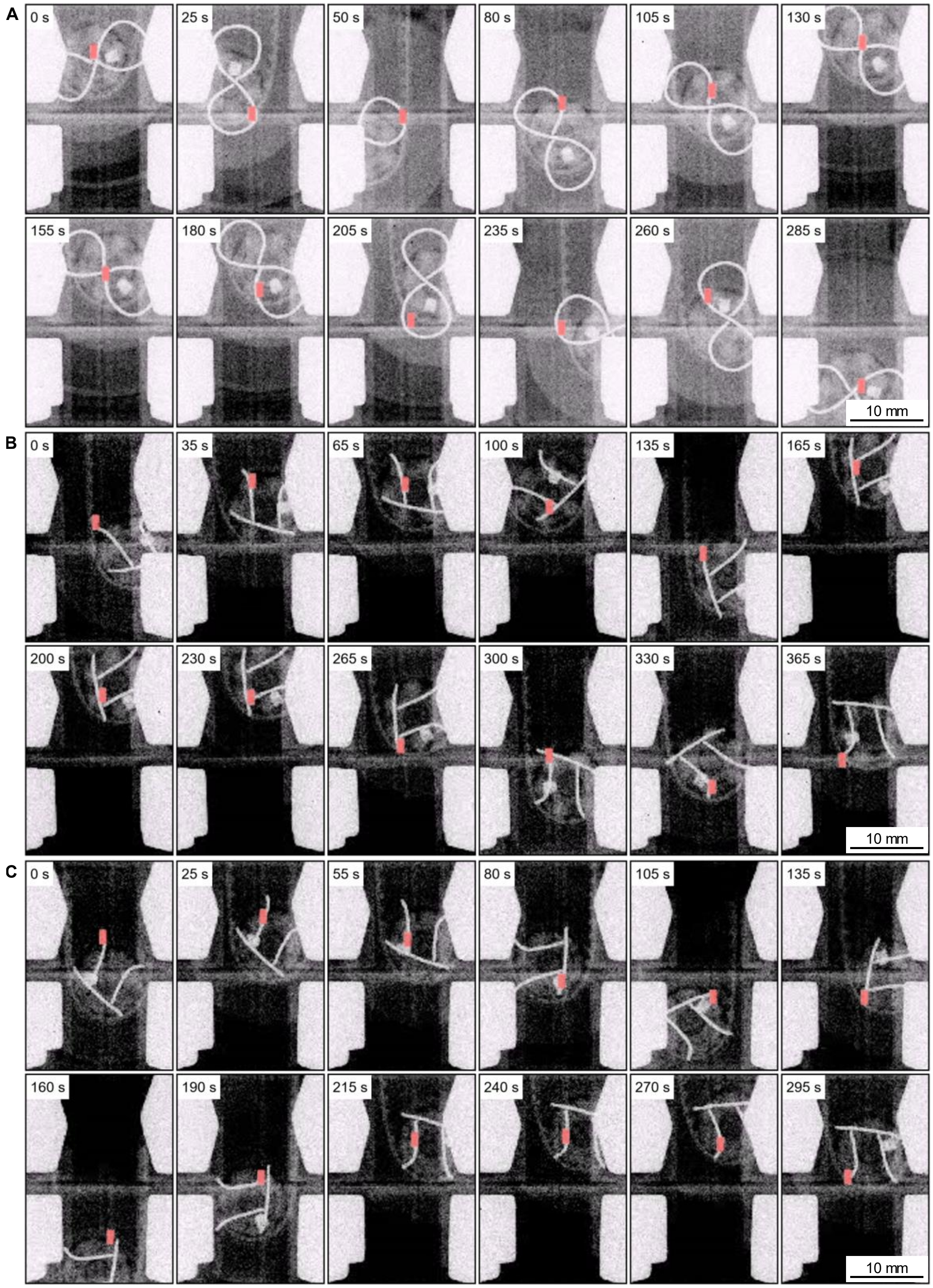

Fig. S6. X-ray image snaphots of a complete path following experiment on ∞-shaped and π-shaped paths inside a fresh porcine brain. Red rectangle represents the robot, where the desired paths are visible in the X-ray images using copper wires bent in the shape of the desired paths and located under the brain tissue.

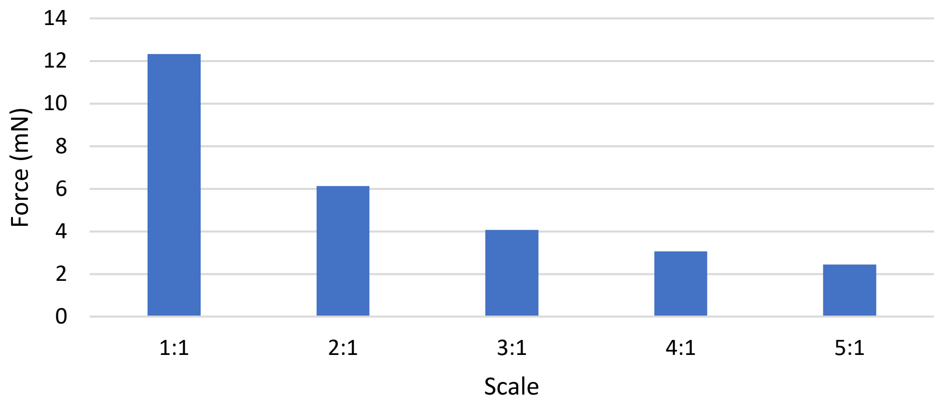

Fig. S7. The calculated maximum forces applied to the robot (a cylindrical NdFeB magnet with 1 mm diameter and 2 mm length) when the magnet array is scaled up. The magnitude of the force is inversely proportional to the length scaling factor.

Table S1. The magnetic axes and positions of the magnets in the final magnet array optimal design shown in Fig. 3A. Angle of the magnetic axis is the angle from the *x*-axis towards the *r*-axis. A sketch for the magnet positions and orientations is given in **Fig. S2.**

	x-position (mm)	*r-position (mm)*	*Angle of the magnetic axis (deg)*
**Layer 1**	-24.85	39.62	275.58
**Layer 2**	-10.20	39.35	228.13
**Layer 3**	4.62	40.03	176.89
**Layer 4**	20.05	40.24	140.41
**Layer 5**	35.19	40.13	104.40

Table S2. Path-following errors in three different ∞-shaped (infinity) and π-shaped (pi) path-following experiments in a gelatin soft tissue phantom. Statistical values are calculated over 25-55 (depending on the total duration of the experiments) samples for each path.

	Mean error (mm)	*Maximum error (mm)*	*Standard deviation (mm)*
**Infinity 1**	0.31	0.89	0.26
**Infinity 2**	0.47	1.00	0.30
**Infinity 3**	0.38	0.93	0.29
**Pi 1**	0.39	1.40	0.29
**Pi 2**	0.39	0.78	0.23
**Pi 3**	0.20	0.86	0.18

Table S3. Path-following errors in two different ∞-shaped (infinity) and π-shaped (pi) path-following experiments in *ex-vivo* porcine brain experiments. Statistical values are calculated over 30-75 (depending on the total duration of the experiment) samples for each path.

	Mean error (mm)	*Maximum error (mm)*	*Standard deviation (mm)*
**Infinity 1**	0.30	0.92	0.27
**Infinity 2**	0.29	0.75	0.17
**Pi 1**	0.28	0.91	0.18
**Pi 2**	0.24	1.12	0.28

Supplementary Movies

Movie S1. Open-loop magnetic trapping stability experiments.

Movie S2. Path-following experiments inside a gelatin soft tissue phantom.

Movie S3. *Ex-vivo* path-following experiments inside a fresh porcine brain.
